# Pollen loads of eucalypt and other pollen types in birds in NW Spain

**DOI:** 10.1016/j.dib.2015.09.012

**Published:** 2015-09-30

**Authors:** María Calviño-Cancela, Max Neumann

**Affiliations:** Department of Ecology and Animal Biology, University of Vigo, Faculty of Biology, Experimental Sciences Building, Campus Lagoas Marcosende, E-36310 Vigo, Spain

**Keywords:** Alien species, Bird pollination, *Eucalyptus globulus*, Mutualistic interactions, Naturalization, Novel ecosystems

## Abstract

Here we present the amount of pollen of eucalypt and pollen of other types for birds captured in two bird ringing stations for 14 months (March 2014 to April 2015) in NW Spain. Common and latin names of all birds species captured, together with the number of captured individuals (N), prevalence of eucalypt pollen (percentage of individuals with eucalypt pollen) and of pollen of other types and average pollen loads per individual for eucalypt and other pollen types is presented. See [1] for further information and discussion.

**Specifications Table**

**Table:** 

Subject area	*Biology*
More specific subject area	*Ecology*
Type of data	*Table*
How data was acquired	*Samples taken from bird feathers analyzed under the microscope*
Data format	*Basic statistics calculated*
Experimental factors	*Percentage of individuals with pollen and average pollen loads per individual for each bird species captured*
Experimental features	*Pollen grains attached to the bill and surrounding feathers* (*forehead, chin and cheeks*) *was collected from all individuals captured in mist nets in two ring stations in a* 14-*month period.*
Data source location	*Darbo and Coiro, Spain* (42°15′48.01″N 8°47′46.97″W and 42°16′29.60″N 8°46′16.61″W)
Data accessibility	*All data is in this paper*

**Value of the data**

• Eucalypts are widely planted around the world and are pollinated mainly by birds in its native range. In Europe, where they have been introduced, there are no specialist bird pollinators but eucalypt flowers are visited by opportunistic birds. The establishment of novel pollination interactions is necessary for seed production, establishment and spread in the introduced area.
• These data show the prevalence and strength of the interaction between eucalypts and birds captured in an area where *Eucalyptus globulus* is widely planted.
• This data set provides the first quantitative estimation of the frequency of eucalypt flower use by birds in an area of eucalypt introduction.
• This data set shows what bird species interact with eucalypt flowers, what is the proportion of individuals that use them and what is the relative importance of this resource for birds.

## 1 Data

The data presented here show the prevalence of eucalypt pollen (percentage of individuals with eucalypt pollen) and of pollen of other types and the average pollen loads per individual for eucalypt and other pollen types for all bird species captured in a 14-month period, together with the number of captured individuals. See [1] for further information and discussion.

## 2 Experimental design, materials and methods

The study was carried out in two constant effort bird ringing stations operated by the bird ringing group Anduriña (see acknowledgments) in NW Spain (Darbo: 42°15′48.01″N 8°47′46.97″W; Coiro: 42°16′29.60″N 8°46′16.61″W). The stations are located 2.5 km apart in mixed landscapes with agricultural land, abandoned fields, native forest patches, forestry plantations (of *Eucalyptus globulus* and *Pinus pinaster*) and scattered houses with their gardens. The two sites differ in the surface covered by *E. globulus*. In Coiro, *E. globulus* stands covered 19.5% of the area comprised within 300-m distance from mist nets (total area of 31.3 ha), with a minimum distance between mist nets and E. globulus trees of c. 50 m. In Darbo, *E. globulus* stands covered 7.9% (of an area of 17.7 ha) and the minimum distance between mist nets and *E. globulus* trees was c. 200 m. We set nine 12-m long nets in Darbo, making a total of 108 m arranged in three 36-m long lines. In Coiro, we set six 12-m long nets, making a total of 76 m arranged in four lines of 12 m or 24 m. Mist nets were operated one day per month in each site, from dawn to dusk, usually on two consecutive Saturdays: the closest to the 15th day of each month and the next one (weather permitting, avoiding rainy and windy days). The study was carried out between March 2014 and April 2015 (i.e. 14 months). For all birds captured, we collected pollen grains attached to the bill and surrounding feathers (forehead, chin and cheeks). For this, we used a lab spatula impregnated with a glycerine-based gel, which was then transferred to microscope slides [2]. When the amount of pollen was too much for one slide, we used two slides, or transferred a subsample of the whole amount to slides (1/2 or 1/4). Samples were processed by acetolysis [3] to aid pollen identification. The sampling effort was adjusted according to the density of pollen in each slide: for slides with low pollen density (≤2500 grains/cm^2^) pollen was quantified in 30 fields at ×100 magnification (19% of the area sampled; [4]), with further observation of the pollen grains detected in each field at ×400 magnification for identification. For slides with high pollen density (>2500 grains/cm^2^) we used 30 fields at ×400 magnification. Pollen was identified using our own pollen reference collections and the aid of expert palynologists (see acknowledgments).
