# Supplementary material for: Pollen loads of eucalypt and other pollen types in birds in NW Spain
Source: Data Brief. 2015 Sep 30;5:348–50. doi: 10.1016/j.dib.2015.09.012 (PMC4602354; doi:10.1016/j.dib.2015.09.012)
Supplement: Supplementary file 3 — Supplementary material [file mmc3.doc]

Data in Brief

María Calviño-Cancela and Max Neumann. Pollen loads of eucalypt and other pollen types in birds in NW Spain.

Table 1: Common and latin names of all birds species captured, together with the number of captured individuals (N), prevalence of eucalypt pollen (percentage of individuals with eucalypt pollen) and of pollen of other types and average pollen loads per individual for eucalypt and other pollen types.

| Common name | Latin name | N | Prevalence- eucalypt | Prevalence- others | Pollen load- eucalypt | Pollen load-others |
| --- | --- | --- | --- | --- | --- | --- |
| European Turtle Dove | *Streptopelia turtur* | 1 | 0 | 0 | 0 | 0 |
| European Nightjar | *Caprimulgus europaeus* | 1 | 0 | 100 | 0 | 10.8 |
| Eurasian Wren | *Troglodytes troglodytes* | 29 | 10.3 | 37.9 | 0.5 | 6 |
| Dunnock | *Prunella modularis* | 19 | 10.5 | 36.8 | 1.7 | 13.8 |
| European Robin | *Erithacus rubecula* | 73 | 17.8 | 38.4 | 12.5 | 12.6 |
| Common Nightingale | *Luscinia megarhynchos* | 5 | 0 | 20 | 0 | 1 |
| Common Blackbird | *Turdus merula* | 57 | 19.3 | 56.1 | 1.8 | 7.6 |
| Song Thrush | *Turdus philomelos* | 13 | 30.8 | 15.4 | 3.2 | 7.3 |
| Cetti's Warbler | *Cettia cetti* | 9 | 11.1 | 0 | 0.6 | 0 |
| Zitting Cisticola | *Cisticola juncidis* | 2 | 0 | 50 | 0 | 2.6 |
| Common Grasshopper Warbler | *Locustella naevia* | 1 | 0 | 100 | 0 | 5.2 |
| Melodious Warbler | *Hippolais polyglotta* | 4 | 0 | 100 | 0 | 18.3 |
| Sardinian Warbler | *Sylvia melanocephala* | 15 | 26.7 | 33.3 | 3.5 | 2.1 |
| Common Whitethroat | *Sylvia communis* | 3 | 0 | 33.3 | 0 | 3.5 |
| Eurasian Blackcap | *Sylvia atricapilla* | 116 | 62.9 | 72.4 | 121544.9 | 986 |
| Common Chiffchaff | *Phylloscopus collybita* | 25 | 84 | 68 | 17086.4 | 115.4 |
| Willow Warbler | *Phylloscopus trochilus* | 1 | 0 | 0 | 0 | 0 |
| Common Firecrest | *Regulus ignicapilla* | 7 | 28.6 | 28.6 | 10983.8 | 19.5 |
| European Pied Flycatcher | *Ficedula hypoleuca* | 2 | 0 | 50 | 0 | 5.2 |
| Long-tailed Bushtit | *Aegithalos caudatus* | 11 | 81.8 | 27.3 | 19689.2 | 2.4 |
| European Crested Tit | *Lophophanes cristatus* | 1 | 100 | 100 | 2551.7 | 209.6 |
| Coal Tit | *Periparus ater* | 12 | 41.7 | 91.7 | 1114.3 | 7725.5 |
| Blue Tit | *Cyanistes caeruleus* | 20 | 55 | 65 | 159 | 88.6 |
| Great Tit | *Parus major* | 18 | 33.3 | 55.6 | 1783.5 | 376.4 |
| Short-toed Treecreeper | *Certhia brachydactyla* | 5 | 20 | 80 | 2.1 | 9.4 |
| Eurasian Jay | *Garrulus glandarius* | 4 | 25 | 25 | 1.3 | 2.6 |
| Eurasian Magpie | *Pica pica* | 1 | 0 | 100 | 0 | 10.5 |
| House Sparrow | *Passer domesticus* | 7 | 0 | 42.9 | 0 | 51.6 |
| Common Waxbill | *Estrilda astrild* | 6 | 0 | 33.3 | 0 | 1.7 |
| Common Chaffinch | *Fringilla coelebs* | 3 | 0 | 66.7 | 0 | 5.2 |
| European Serin | *Serinus serinus* | 3 | 66.7 | 66.7 | 31.4 | 31.4 |
| European Greenfinch | *Carduelis chloris* | 5 | 0 | 60 | 0 | 11.5 |
| Eurasian Bullfinch | *Pyrrhula pyrrhula* | 6 | 16.7 | 50 | 0.9 | 7004.6 |
